# High-Precision Fabrication of Micro Monolithic Tungsten Ball Tips via Arc Discharge and the Taguchi Method

**DOI:** 10.3390/mi12091042

**Published:** 2021-08-29

**Authors:** Zhen-Ying Cheng, Pan Yao, Yong-Jun Wang, Chen Chen, Li-Juan Chen, Rui-Jun Li, Qiang-Xian Huang

**Affiliations:** 1 School of Instrument Science and Optoelectronic Engineering, Hefei University of Technology, Hefei 230009, China; chengzhenying01@hfut.edu.cn (Z.-Y.C.); 2020110010@mail.hfut.edu.cn (P.Y.); wangyongjun@mail.hfut.edu.cn (Y.-J.W.); chenchenhfut@yeah.net (C.C.); chenlj@hfut.edu.cn (L.-J.C.); huangqx@hfut.edu.cn (Q.-X.H.); 2 Anhui Province Key Laboratory of Measuring Theory and Precision Instrument, Hefei University of Technology, Hefei 230009, China

**Keywords:** tungsten ball tip, arc discharge, Taguchi method, process parameters

## Abstract

A micro ball tip is a core component of high precision coordinate measuring machines. The present micro ball tips cannot satisfy the high-precision measuring requirements of high aspect ratio microstructures due to their large diameter and low accuracy. In the previous study, we fabricated a micro monolithic tungsten ball tip by using arc discharge and surface tension principles. However, the fabrication success rate of forming a micro ball tip is less than 10%. In the present study, the Taguchi method has been applied to increase the fabrication success rate, and it has increased to 57.5%. The output response is evaluated in terms of the diameter, roundness, and center offset of the tungsten probe ball tips. The smaller-the-better signal-to-noise ratio is applied to analyze the influence of various parameters. The proposed parameters can be used to increase the fabrication success rate and accuracy of the monolithic probe ball tip.

## 1. Introduction

Various micro devices, such as fuel nozzle, ceramic bearing, and optical fiber ferrule, have emerged with the rapid development of ultraprecision machining technology. These devices have high aspect ratio micro features and micrometer level precision requirements. The traditional noncontact measurement method cannot satisfy the requirements due to the low lateral resolution and limited longitudinal measurement range [1,2,3]. A high precision coordinate measuring machine (CMM) equipped with micro ball tips is required urgently. The micro ball tips’ diameter should be less than 100 μm; the smaller the roundness and center offset errors, the better.

In recent years, several materials have been used to fabricate micro ball tips. Yang P et al. fabricated a micro ruby ball, with a diameter of 120 μm and roundness and center offset errors of more than 1 μm. However, the phenomenon of adhesive wear occurs when the ruby ball contacts with a surface of aluminum or cast iron, thereby leading to measurement errors [4]. Ito et al. fabricated a glass fiber ball with a minimum diameter of 25 μm, a roundness error of 6 μm, and a center offset error of 3 μm. The glass fiber is easily melted into small balls. However, the balls and rods are easily damaged because of their high brittleness [5]. Peggs et al. fabricated a silicon ball, with a diameter of 300 μm and roundness of 1 μm. Silicon nitride has high hardness. However, it is vulnerable to wear when contacted with steel materials [6,7]. Tungsten is a suitable material for fabricating micro ball tips because of its high hardness and wear resistance [8,9]. Three methods, namely, adhesion, material removal, and melting, are used to fabricate micro ball tips. For adhesion, the center offset error is difficult to control because of the small diameter of the ball. Spilled adhesive affects the roundness and finish of the ball during bonding [10,11]. Material removal has the shortcomings of complex experimental equipment and a coarse surface of the ball [12,13]. 

The authors proposed and fabricated micro monolithic tungsten ball tips through melting [14]. The fabricated monolithic tungsten ball tips have the advantage of high precision, are easy to operate, and have a low cost. The geometry of the sphere can be monitored with a vision system in the optical image system. The fabrication success rate is affected by several process parameters, such as impulse voltage, discharge duration, spacing between electrodes, pulse frequency, and pulse width. The Taguchi method is used in the present study. The fabrication success rate increases from less than 10% to 57.5%. The effects of discharge parameters on roundness and eccentricity are presented. The images of the surface morphology of the tungsten ball tip are shown. These results can be used to increase the fabrication success rate and accuracy of the monolithic probe ball tip. 

## 2. Principles, Setups, and Process Parameter Ranges

### 2.1. Principles

**Figure 1:**
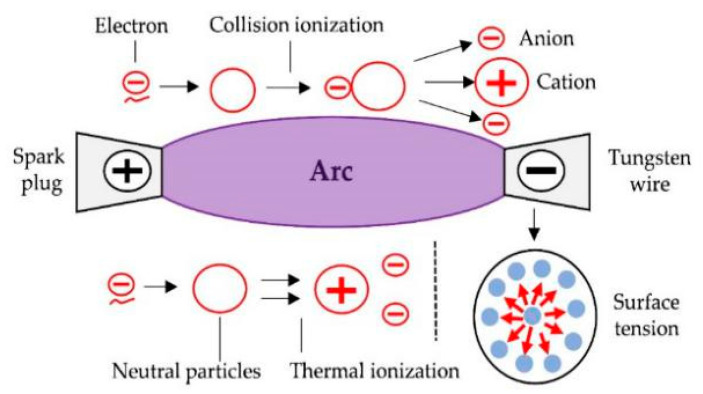
Schematic diagram of arc and surface tension.

The principle of discharge is shown in Figure 1. Tungsten wire is used as the cathode, and a spark plug is used as the anode. An arc is generated when sufficient voltage is applied to the two electrodes. The atoms at the discharge point vaporize and emit charged particles in plasmatic condition. The electrons and ions that make up the gas acquire energy to accelerate the movement, and the temperature rises sharply. The accelerated electrons and neutral molecules collide with each other to strengthen the vibration motion of molecules and increase the temperature of gas. The temperature of the tungsten wire rises sharply until it melts. Each atom is evenly pulled out by adjacent liquid atoms in each direction when the tungsten wire melts. The molten tungsten becomes a tungsten ball tip under the action of liquid surface tension [15,16]. 

### 2.2. Experimental Setups

**Figure 2:**
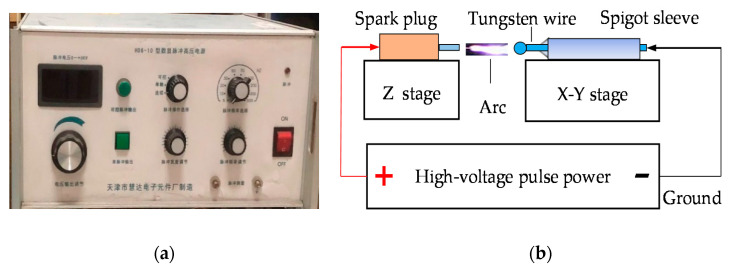
Diagram of the experimental device. (**a**) Experimental device and (**b**) high-voltage pulse power.

A high-voltage pulse power (HD6-10, Tianjin Huida Electronic Factory, Tianjin, China) was used. The process parameters of the power, such as impulse voltage, frequency, pulse width, and discharge duration, were continuously adjustable, as shown in Figure 2. A Z stage and X-Y stage were used to align the two electrodes and change the space between them. The diameter of tungsten wire was 100 μm, and the purity was 99.99%. The shape of the tungsten wire tip can affect the properties of the tungsten ball. We maintained flat and smooth tungsten wire tip by fast cutting. We observed the shape of the tungsten wire tip through a magnifying glass to maintain its state.

### 2.3. Ranges of the Process Parameters

The diameter, roundness error, and center offset error are the key parameters of tungsten ball tips. These parameters are affected by discharge duration, spacing between electrodes, impulse voltage, discharge frequency, and pulse width. Determining the appropriate operating range for each parameter is necessary to obtain the best combination of process parameters.

#### 2.3.1. Spacing between Electrodes

According to Paschen’s law, the breakdown voltage of a uniform electric field in air is approximately linearly related to the electrode spacing [17]. When the pulse voltage exceeds the breakdown voltage, an arc is generated. In other words, when the electric field strength between the spark plug and the tungsten wire exceeds the critical value (about 3 × 10^6^ V/m), the electric field force causes the electrons on the cathode surface to escape and become free electrons in space [18,19]. The free electrons collide with the neutrons in space and form free ions, thus greatly increasing the electrical conductivity and generating an electric arc. Therefore, this relationship can be expressed as follows:

(1) $$E=\frac{U}{d}>3\times 10^{6}\;V/m$$

where *E* is the uniform electric field intensity, *U* is the pulse voltage, and *d* is the spacing between electrodes.

The highest voltage of our pulse power supply was 10 kV (the pulse frequency was 0–300 Hz, and the output current was 10 mA). The impulse voltage was 10 kV in this study to obtain high discharge energy. The maximum spacing between electrodes should be less than 3.3 mm. Hence, 1, 1.5, and 2 mm were the typical values of spacing between electrodes.

#### 2.3.2. Discharge Parameters

**Figure 3:**
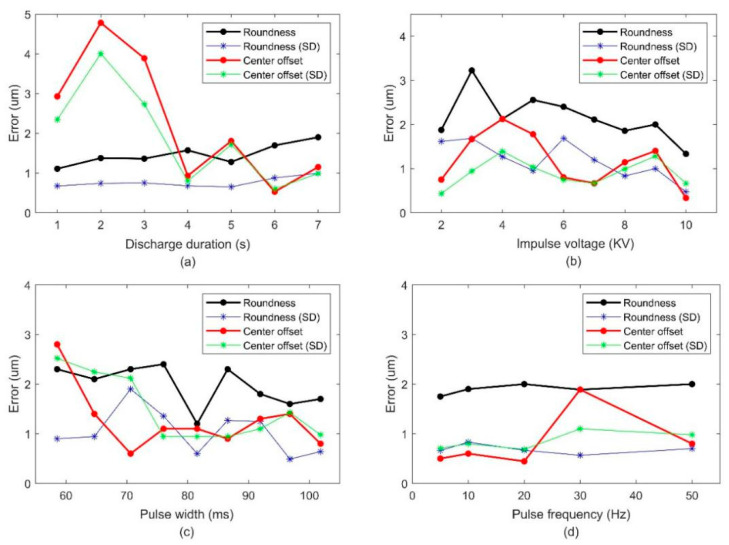
Relationship between the roundness error, center offset error, and process parameters. (**a**) Discharge duration, (**b**) impulse voltage, (**c**) pulse width, and (**d**) pulse frequency.

The influence of each single variable of the micro ball tip was studied, and the appropriate range of a single parameter is obtained, depending on the control variable method [20]. The parameters used in each group control variable experiment are shown in Table 1. The experiments for each process parameter were repeated 10 times, the roundness and center offset errors of the tungsten ball tips were measured, and the average values were used. The relationships between the process parameters and roundness and center offset errors are shown in Figure 3. The process parameters leading to small roundness and center offset errors were selected. Therefore, 10 kV was determined as the best impulse voltage. The pulse frequencies were 5, 10, and 20 Hz, the time intervals were 5, 6, and 7 s, and the pulse widths were 81.6, 91.9, and 97.8 ms.

## 3. Optimization of Process Parameters and Results

The Taguchi method was founded in the early 1970s by Dr. Taguchi, a famous Japanese quality control expert. This method is a technology mainly used to improve experimental efficiency, reduce costs, and improve product quality in the early stage of design and product development [21,22]. The parameter design approach proposed by the Taguchi method was adopted to increase the fabrication success rate and accuracy of the monolithic probe ball tip. 

Figure 3b shows that the roundness and center offset errors of tungsten ball were smaller than the others when the impulse voltage was 10 kV. Therefore, the impulse voltage was 10 kV to reduce the complexity of Taguchi experiments. On the basis of the control variable experiments, four influential fabricating factors were selected, including discharge duration (s), spacing between electrodes (mm), pulse frequency (Hz), and pulse width (ms). 

### 3.1. Taguchi Experiments

To evaluate these factors, they were considered with three levels. The involved factors and their levels are shown in Table 2. The orthogonal table of L9 (34) was used to design the test parameters. The parameters of each factor were filled into the orthogonal table in accordance with the rules, as presented in Table 3.

The signal-to-noise (S/N) ratio is the pointer to a robust design and shows the sensitivity of the product function to the noise factor. Therefore, it was applied in the Taguchi method as the quality characteristic of choice [23,24]. A smaller-the-better type characteristic was used as the objective function (Equation (2)) in this experiment to achieve small diameter, better sphericity, small eccentricity, and high fabrication success rate.

(2) $$\eta =-10\log\left(\frac{1}{n}\sum_{i=1}^{n}{y_{i}}^{2}\right)$$

where *η* is the S/N ratio obtained through the calculation of experimental observation, *yi* can be the diameter, roundness, and center offset of probe tips, and *n* is the experimental frequency.

In accordance with the experimental requirements of orthogonal array, nine groups of experiments were conducted and repeated 10 times for each group. The average value of each characteristic parameter was considered. The mean values of the probe’s diameter, roundness, and center offset are shown in Table 3. The values of η were calculated and shown in Table 4.

### 3.2. Mean Value Analysis

**Figure 4:**
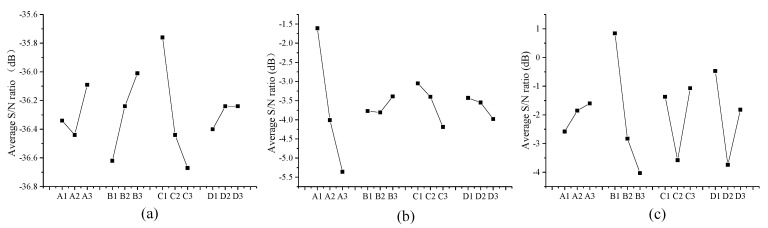
Average S/N ratio responses for the probe tip parameters: (**a**) diameter, (**b**) roundness, and (**c**) center offset.

The mean values of the S/N ratio in the same level of each factor were calculated in accordance with Table 2 and Table 3 to find the optimal level combination for the minimum diameter, roundness, and center offset. The mean S/N ratio for roundness at level 1 of factor B was calculated as follows:

(3) $$\bar{\eta_{\mathrm{dB}1}}=\frac{1}{3}\left(\eta_{d}\left(1\right)+\eta_{d}\left(4\right)+\eta_{d}\left(7\right)\right)$$

where *d* and *B* in the subscript denote the diameter and factor B, respectively. Factor B is set to level two only in experiments 1, 4, and 7. The mean S/N ratios for all levels of factors can be obtained in a similar approach, as shown in Table 5. Figure 4 shows the S/N responses for the probe diameter, roundness, and center offset.

The S/N ratios were close enough to zero to reduce the volatility of tungsten wire melting into a ball. Figure 4 can be used to determine the preliminary combination of experimental parameters for preparing the following research by variance analysis. The best diameter can be obtained with the experimental combination of (A3, B3, C1, and D2). The minimum roundness error can be obtained with the experimental combination of (A1, B3, C1, and D1). The minimum center offset error can be obtained with the experimental combination of (A3, B1, C3, and D1).

### 3.3. Variance Analysis

Variance analysis was used to analyze the influence percentage of each factor on the experimental results. This process was performed to quantitatively investigate the effects of the forming parameters. In this analysis, every best characteristic of the tungsten probe ball tips can be fabricated by different combinations of parameters. However, the selection of some parameters is contradictory. Therefore, finding an experimental combination of parameters that can consider all the probe characteristics by using the three-existing combination of experimental parameters is important.

(4) $${\mathrm{SS}}_{VF}=3{\sum_{i=1}^{3}\left(\bar{\eta_{VFi}}-\bar{\eta_{V}}\right)}^{2}$$

where *V* denotes the “variable”, which can be the diameter, roundness, or center offset. *F* denotes the “Factor”, which can be A, B, or C. *i* is the sequence number of the factor’s level. $\bar{\eta_{V}}$ and $\bar{\eta_{VFi}}$ can be found in Table 2 and Table 3, respectively.

Minitab software was used for analysis of variance. A quantitative understanding of the factor effect by ANOVA is presented as follows. In Table 6, it is observed that the discharge duration, spacing between electrodes, pulse frequency, and pulse width significantly influenced the roundness error. In Table 7, the center offset error was significantly influenced by spacing between electrodes, pulse frequency, and pulse width (at 95% confidence level). However, the effect of discharge duration on eccentricity was significant at 85%, which can be considered as a secondary factor in the manufacturing process. In Table 8, the contribution percentage shows that the most significant parameter influencing the probe diameter was the pulse frequency, the critical factor affecting the probe roundness was the discharge duration, and the important factor influencing the probe center offset was the spacing between electrodes. The S/N ratios and variance analysis indicate that the best of the nine parameter combinations were A1 (5 s), B1 (1 mm), C1 (5 Hz), and D1 (81.6 ms), considering the importance of the factors affecting the probe roundness, the center offset, and that the mean S/N ratio of them were closer to zero.

### 3.4. Optimum Parameters and Confirmation Experiments

An image measuring instrument (ADI-ORS100, Nanjing Optics Robot Technology Corp, China; Resolution: 1 um) was used to measure the diameter, the roundness error, and center offset error of the fabricated probe balls. The measurement results can be viewed through the software matched with the instrument. Compared with the fitting circle, the difference between the maximum radius and the minimum radius of the tungsten ball tip contour is the roundness error [25]. The diameter of the fitting circle is the diameter of the tungsten ball tip. The distance from the center of the fitted circle to the centerline of the probe is the center offset error.

**Figure 5:**
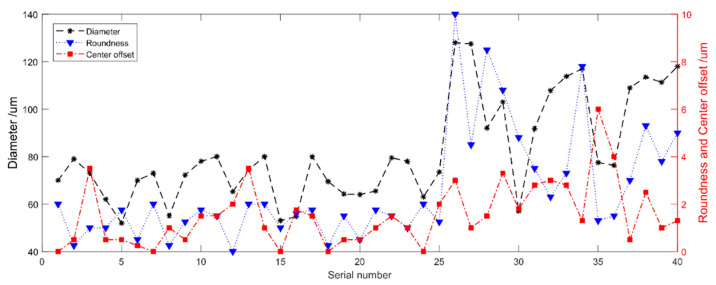
Measuring results of the fabricated ball tips.

The confirmation experiment was carried out at the optimum setting of the significant forming parameters. This strategy was applied to fabricate a number of micro probes. A total of 40 experiments were conducted 40 times in accordance with the obtained combination of experimental parameters. The diameter, roundness error, and center offset error of these probe ball tips were measured three times in four view angles with the image measurement instrument, and their mean values were obtained. Therefore, the decimals appeared, although the resolution of the optical image system was 1 µm. The mean measurement values of the 40 tungsten ball tips, which are shown in Figure 5, were used to represent the ball tip’s diameter, center offset, and roundness. The diameters of the tungsten probe balls were between 52 and 127.5 μm. The roundness and center offset errors of these probe ball tips were between 0 and 6 μm. We placed the ball tips with high precision in the left and the others in the right. A total of 57.5% of the fabricated tungsten ball tips had a roundness and a center offset error of less than 2 μm, and 18% of them had a roundness and a center offset error of less than 1 μm.

**Figure 6:**
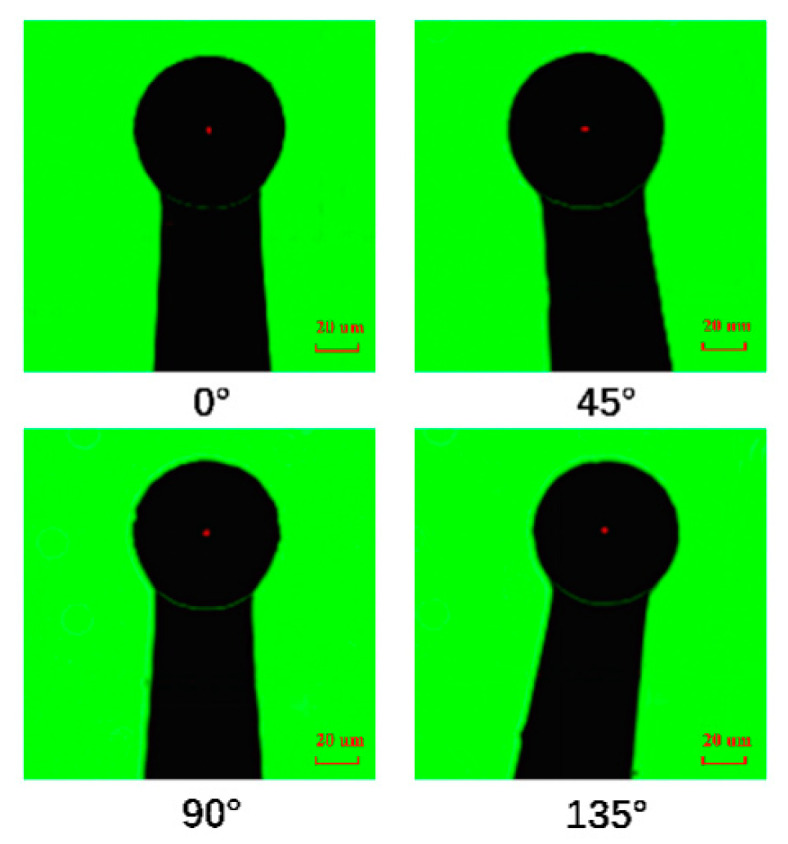
Images of the tungsten ball tip at four viewed angles.

**Figure 7:**
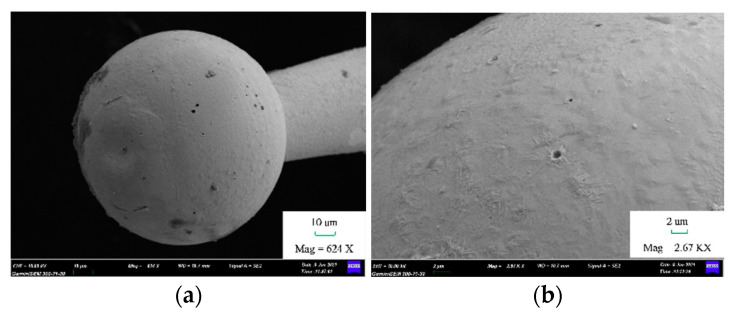
Image of tungsten probe ball under scanning electron microscope. (**a**) Tungsten probe ball and (**b**) local of tungsten probe ball.

**Figure 8:**
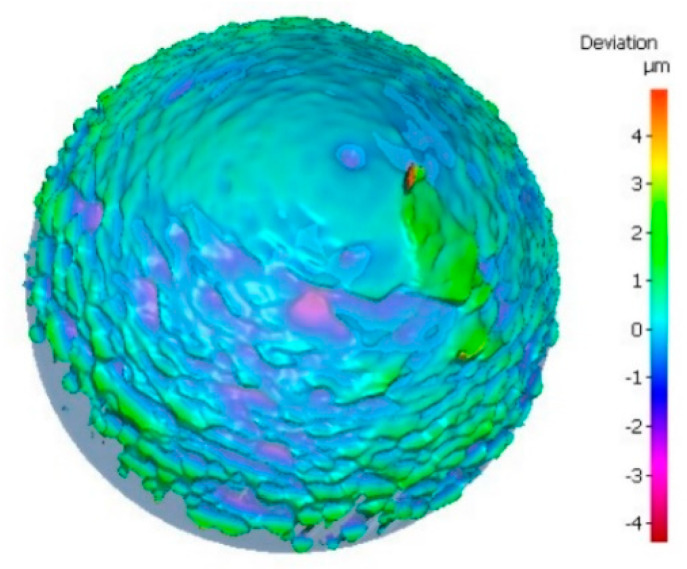
Image of roughness measurement.

The roundness and center offset of 23 tungsten ball tips were less than 2 μm. The average roundness of these tungsten ball tips was 1.2 μm, and the standard deviation was 0.64 μm. The average center offset of these tungsten ball tips was 0.8 μm, and the standard deviation was 0.66 μm. One of ball tips at four viewed angles is shown in Figure 6. The diameter was 69.5 μm, the roundness was 0.3 μm, and the center offset was 0.1 μm. Another ball tip was analyzed by field emission scanning electron microscope (FESEM) and by a high precision 3D shape detector. The model of the instrument was Zeiss Gemini SEM 300 and Alicona. The diameter was 98 μm, as shown in Figure 7a. Figure 8 shows that the surface of tungsten ball probe tip was smooth and clean and the mean roughness deviation was 0.537 μm.

### 3.5. Significance Test Result

The significance test was carried out to investigate the difference before and after parameter optimization. The following steps should be carried out.

The variance of roundness and center offset before and after optimization should be calculated.

(5) $$\begin{array}{l} \sigma_{x}^{2}=\frac{1}{n_{x}}{\sum \left(x_{i}-\bar{x}\right)}^{2}\\ \sigma_{y}^{2}=\frac{1}{n_{y}}{\sum \left(y_{i}-\bar{y}\right)}^{2} \end{array}$$

where $x_{i}$ indicates the roundness and center offset before optimization and $y_{i}$ indicates the roundness and center offset after optimization. $\bar{x}$ and $\bar{y}$ are their average. $n_{x}$ and $n_{y}$ denote the numbers of tungsten balls, and they are both 40.

A *t*-test was used, and the significant factor was α = 0.05. If the *t*-value of roundness and center offset satisfies *t* < $t_{\alpha }$ ($n_{x}+n_{y}-2$), then the result is insignificant; otherwise, it is significant. In addition, the higher value is more significant.

(6) $$t=(\bar{x}-\bar{y})\sqrt{\frac{n_{x}n_{y}\left(n_{x}+n_{y}-2\right)}{\left(n_{x}+n_{y})(n_{x}\sigma_{x}^{2}+n_{y}\sigma_{y}^{2}\right)}}$$

After calculation, the *t*-value of roundness was 2.183, and the *t*-value of center offset was 3.754. $t_{\alpha }$ is shown in the table, that is, 1.665. We found that the *t*-values of roundness and center offset were greater than $t_{0.05}\left(78\right)=1.665$. Therefore, the fabrication success rate was significantly improved.

## 4. Discussions

In order to manufacture ball tips with smaller dimension and higher precision in future work, the mechanisms of ball forming caused by the main factors are discussed here.

The arc energy accumulation was proportional to the pulse frequency. The plasma changed from primary ionization to secondary ionization, and the number and velocity of particle collisions increased sharply due to the accumulation of the energy in space. In macroscopic view, the arc temperature rose sharply, more of the tungsten wire gasified, and the diameter of the tungsten ball became smaller.

The longer the discharge time was, the longer the tungsten wire remained in the molten state. Because of the surface tension, it tended to form a sphere with small roundness errors.

With the increase in the electric step spacing, the arc could not hit the center of the tungsten wire. As a result, the center of the circle shifted and the eccentricity increased. Therefore, the electric step spacing should be controlled and reduced.

The temperature gradient of arc plasma was speculated to be large and the heat transfer is uneven, resulting in the crater on the surface of the tungsten ball. The tungsten wire was oxidized in the air to form small particles, which were hit by the arc on the molten tungsten ball. Therefore, the temperature gradient of arc plasma should be small during the manufacturing process in order to make the tungsten balls smooth.

## 5. Conclusions

Uniform electric field intensity and control variable experiments were performed to obtain the appropriate range of the process parameters to promote the success rate of fabricating micro monolithic tungsten ball tips using arc discharge. The output response of the parameters was evaluated in terms of the diameter, roundness error, and center offset error of the probe tips. The parameters were optimized using the Taguchi method. On the basis of the S/N ratio and variance analyses, the best process parameters for the required ball diameter, roundness, and center offset were obtained, namely, impulse voltage (10 kV), discharge duration (5 s), spacing between electrodes (1 mm), pulse frequency (5 Hz), and pulse width (81.6 ms). A total of 57.5% or 18% of the fabricated tungsten ball tips had a roundness and a center offset error of less than 2 μm or 1 μm using the obtained optimum parameters. The prepared probe ball tips can be used to detect the microstructures with high aspect ratio features. In future, we intend to use a strong magnetic field to reduce the effect of gravity on the center offset, to study the preparation of tungsten spheres in argon atmosphere to obtain a more polished surface, and to deduce the mathematical-physical model of tungsten balls fabrication. Additionally, optimization algorithms, such GRA or PSO, will be further studied to improve the quality of ball manufacturing.

## Figures and Tables

**Table 1:** Experimental parameters of each group control variable experiment.

Controlled Variables	Experimental Parameters			
	Impulse Voltage (kV)	Pulse Frequency (Hz)	Discharge Duration (s)	Pulse Width (ms)
Impulse voltage (kV)	2–10	5	6	81.2
Pulse frequency (Hz)	3	5–50	6	81.2
Discharge duration (s)	10	5	1–7	81.2
Pulse width (ms)	10	5	6	54.1–101.9

**Table 2:** Impact factors and their levels.

Level	A	B	C	D
	Discharge Duration (s)	Spacing between Electrodes (mm)	Pulse Frequency (Hz)	Pulse Width (ms)
1	5	1	5	81.6
2	6	1.5	10	91.9
3	7	2	20	97.8

**Table 3:** Orthogonal array of L9 (34).

L9	A	B	C	D	Discharge Duration (s)	Spacing between Electrodes (mm)	Pulse Frequency (Hz)	Pulse Width (ms)
1	1	1	1	1	5	1	5	81.6
2	1	2	2	2	5	1.5	10	91.9
3	1	3	3	3	5	2	20	97.8
4	2	1	2	3	6	1	10	97.8
5	2	2	3	1	6	1.5	20	81.6
6	2	3	1	2	6	2	5	91.9
7	3	1	3	2	7	1	20	91.9
8	3	2	1	3	7	1.5	5	97.8
9	3	3	2	1	7	2	10	81.6

**Table 4:** Experimental results and S/N ratios for diameter, roundness, and center offset.

Number	Average Diameter (μm)	ηd (dB)	Average Roundness (μm)	ηr (dB)	Average Center Offset (μm)	ηc (dB)
1	63.25	–36.24	1.03	–0.89	0.59	2.44
2	68.31	–36.39	1.08	–1.40	1.50	–6.70
3	65.19	–36.39	1.28	–2.53	1.16	–3.48
4	70.97	–36.87	1.60	–4.19	0.98	–0.39
5	67.75	–36.87	1.58	–4.81	0.88	–0.19
6	62.62	–35.57	1.38	–3.02	1.45	–4.97
7	67.13	–36.75	2.00	–6.23	0.70	–0.46
8	58.63	–35.47	1.78	–5.23	0.78	–1.60
9	63.34	–36.07	1.48	–4.61	1.33	–3.65

**Table 5:** Mean values in the same level of each factor and variable.

Response Variables	Level	Mean S/N Ratio			
		A	B	C	D
Diameter	1	−36.34	−36.62	−35.76	−36.40
	2	−36.44	−36.24	−36.44	−36.24
	3	−36.09	−36.01	−36.67	−36.24
Roundness	1	−1.61	−3.77	−3.05	−3.43
	2	−4.01	−3.81	−3.40	−3.55
	3	−5.36	−3.39	−4.219	−3.98
Center offset	1	−2.58	0.84	−1.37	−0.47
	2	−1.85	−2.83	−3.58	−3.74
	3	−1.60	−4.03	−1.07	−1.82

**Table 6:** ANOVA for the roundness error.

Parameter	DOF	SS	MS	F	P
Discharge duration (s)	2	0.83297	0.41649	367.49	0.0003
Spacing between electrodes (mm)	2	0.05003	0.02501	22.07	0.0161
Pulse frequency (Hz)	2	0.12213	0.06106	53.88	0.0045
Pulse width (ms)	2	0.06521	0.03261	28.77	0.011
Residual	3	0.0034	0.00113		
Total	11	1.1259			

Where SS is the sum of squares of deviation from mean and MS is the mean square.

**Table 7:** ANOVA for the center offset error.

Parameter	DOF	SS	MS	F	P
Discharge duration (s)	2	0.05069	0.02535	3.66	0.1569
Spacing between electrodes (mm)	2	0.54531	0.27265	39.32	0.007
Pulse frequency (Hz)	2	0.25097	0.12549	18.1	0.0212
Pulse width (ms)	2	0.13068	0.06534	9.42	0.0509
Residual	3	0.0509	0.00693		
Total	11	1.21103			

**Table 8:** Variance analysis of characteristic parameters and its contribution.

Control Factors	Diameter		Roundness		Center Offset	
	SSD	%	SSR	%	SSC	%
A	0.19	8.67	21.65	87.87	1.56	2.31
B	0.58	26.60	0.33	1.35	38.57	57.04
C	1.35	62.49	2.16	8.76	11.29	16.69
D	0.05	2.24	0.50	2.02	16.20	23.96

## Abbreviations

The following abbreviations are used in this manuscript:

CMM	Coordinate measuring machine
STB	Smaller-the-better
